# Transplantation Tolerance Induced in Humans at the Fetal or the Neonatal Stage

**DOI:** 10.1155/2011/760319

**Published:** 2011-08-18

**Authors:** Jean-Louis Touraine, Kamel Sanhadji

**Affiliations:** ^1^Claude Bernard University and Hospices Civils de Lyon, France; ^2^Transplant Unit, Pav. P., Hôpital Ed. Herriot, 69437 Lyon Cedex 3, France

## Abstract

Patients transplanted with HLA-mismatched stem cells from fetal livers develop transplantation tolerance to donor antigens. Engraftment needs no conditioning regimen prior to transplantation in neonates with severe combined immunodeficiency disease or in human fetal patients having not yet developed any immune maturity, especially T-cell differentiation. The chimeric patients have donor-derived T lymphocytes which progressively demonstrate positive interactions with other host cells. They also can be shown to be tolerant toward both host and donor antigens. The latter tolerance relies upon clonal deletion from the T-cell repertoire, and it results from the contact between thymocytes of donor origin and dendritic cells or macrophages also deriving from donor stem cells. The former tolerance does not imply clonal deletion of T-cells with host reactivity. Numerous T-cells recognizing the allogeneic, host-type antigens are identified in these patients, but these cells are anergized, following interaction with epithelial cells of the host thymus. Induction of transplantation tolerance at the fetal stage requires minimal engraftment only; in the future it will be possible to further amplify the clinical benefit, using additional cell transplants after birth.

## 1. Introduction

Following the pioneering and most promising work of Billingham, Brent, and Medawar in newborn mice [1], many experimental and some clinical studies have focused on means to induce transplantation tolerance. In human patients, full tolerance has yet been developed in two circumstances only: (a) when the transplant recipient is not immunologically competent or mature and (b) when the transplant involves full replacement of host lymphocytes by donor lymphocytes. The latter condition is obtained by myeloablation and immunosuppression, followed by stem cell transplantation (SCT) [2]. The former mode of tolerance induction is seen in patients with severe combined immunodeficiency disease (SCID) treated with SCT [3] or in fetal patients subjected to SCT prior to their immunological maturation [4].

We review herein our results in infants and human fetuses who were treated by fetal liver SCT and who developed full tolerance to both donor and host antigens [5–13].

## 2. Patients, Materials, and Methods

Nineteen patients with SCID, including 17 infants and 2 fetuses, received fetal liver SCT [4, 7]. Fetal livers were obtained from dead human fetuses under conditions approved by the French National Committee for Bioethics. Donors were aged less than 14 weeks after fertilization. A cell suspension was prepared, cell viability was checked, and cells were administered by intravenous or intraperitoneal injection. In the two fetal recipients with SCID, the cells were injected into the umbilical vein in utero [4]. Fourteen of the 19 patients had evidence of donor cell engraftment and developed immunological reconstitution. All were subjected to immunological investigations, especially on peripheral blood lymphocytes (PBLs). The studies reported herein mainly concern three children who were analyzed more extensively over a long period of time (21–34 years). 

Three nonimmunodeficient fetuses with various diseases (thalassemia major, Niemann-Pick type A disease and hemophilia A) were also analyzed for at least 2 years after the in utero SCT (which was performed by the intraperitoneal route).

HLA typing was initially carried out on PBL, T-cell clones, and EBV-transformed B-cell lines using a previously described cytotoxicity assay [14] and was confirmed more recently using molecular biological methods.

To analyze responses in mixed leukocyte cultures (MLCs), PBL from the patients were cultured together with a variety of irradiated stimulator cells. The proliferative response of T lymphocytes to these stimulator cells was determined by the degree of tritiated thymidine incorporation [12]. The experiments were performed in triplicate, and the results are expressed as the mean ± standard deviation (SD).

## 3. Results

### 3.1. HLA Typing

Table 1 summarizes the HLA phenotypes (class I and class II) of cells of donor and host origin that were prepared from the three most extensively studied patients, all of whom have had a stable chimerism for many years. They had received fetal liver SCTs from several donors but only the HLA phenotype of the permanently engrafted cells is reported here.

Over the past 34 years, many investigations have been carried out in these three patients. On all occasions, most B-cells and antigen-presenting cells were found to be of host origin while all T-cells were of donor origin. Accordingly, T-cell clones prepared from the PBL of these patients only exhibited the HLA determinants of the donor.

With the exception of patient 2, for whom the HLA-A2 antigen was shared by the donor and the host, all patients had a complete mismatch between host-derived and donor-derived cells. T lymphocytes and B lymphocytes or antigen-presenting cells share no class II determinant and almost no class I HLA antigen, since the former are derived from donor stem cells and the latter from host cells.

### 3.2. Mixed Leukocyte Cultures

After immunological reconstitution had developed in the SCID patients treated postnatally, PBLs were prepared and used as responders while irradiated cells of various origins were employed as stimulators [12]. The 2 patients reported in Table 2 demonstrate an inability to mount a proliferative response to host stimulator cells. In contrast, the T lymphocytes from these patients proliferated readily when stimulated by allogeneic cells or by cells from their parents (Table 2).

Among T-cell clones (e.g., tetanus-toxoid-specific T-cell clones) prepared from patients' PBL, virtually none recognized donor HLA antigens, whereas 15 of 50 clones directly recognized HLA antigens of the host [9] and mounted strong proliferative and cytotoxic responses to host-derived cells [9, 13]. The high frequency of CD8+ host-reactive cells in the chimeric patients was comparable with that of alloreactive cells, in contrast with the lack of cells that reacted with the donors [12].

MLCs were performed in 6 additional patients, including the 2 SCID patients treated at the fetal stage, in utero. The results were comparable with those described above, with a specific lack of proliferative response to host stimulator cells. Again, such an absence of proliferation in MLC did not indicate lack of recognition of host antigens, since some T-cell clones could be shown to be host reactive.

### 3.3. Lymphokine Synthesis and Secretion

The production of lymphokines by host-reactive T-cells from these patients was characterized by high levels of gamma-interferon and, following activation, granulocytic-monocyte-colony-stimulating factor, interleukin-5, and interleukin-2 [12]. Interestingly, no interleukin-4 was produced, irrespective of the mode of activation [12, 13]. Spontaneous secretion of interleukin-10 by the patients' cells was regularly found to be increased [5, 6].

### 3.4. Results in Nonimmunodeficient Patients

We report here 3 human fetal patients who have been treated by in utero SCT at the age of 12–14 weeks postfertilization. After birth, the children were repeatedly studied.

In the thalassemic girl, engraftment was ascertained in blood and bone marrow by the presence of (a) hemoglobin A, (b) cells with the donor Y chromosome, and (c) cells with the donor HLA phenotype. However, the number of donor cells remained limited and tended to decrease with age. At 4 years of age, 0.5–1% of bone marrow cells only expressed simultaneously the CD34 marker and the HLA-A32 phenotype of donor origin. This low chimerism on the long-term was not sufficient to ensure significant clinical benefit, but it suggested maintenance of tolerance to donor antigens.

The two other patients had evidence of donor cell survival, in the absence of immunosuppression at any time, with the prolonged presence of cells with HLA markers of the donor [4]. This engraftment was made possible by the immune immaturity of recipients at 12–14 weeks of fetal age. However the number of donor cells did not increase with time. Actually, it became lower after the first 1 or 2 years. The hemophiliac did not generate any antifactor VIII antibody, suggesting tolerance to this factor, possibly as the result of factor VIII production by donor-derived cells and its presentation to the immune system of the developing fetus.

## 4. Discussion

Because of immune incompetence, SCID patients on the one hand and humans in the early stage of fetal development on the other hand can benefit from engraftment of mismatched stem cells. As a source of stem cells to treat our patients, we have used fetal livers, taking advantage of the relative competitive engraftment superiority of fetal liver cells over adult bone marrow cells, especially in fetal recipients [15].

Despite the lack of HLA antigens shared by donor-derived T lymphocytes and the other cells of the body, efficient immune interactions develop in SCID patients treated pre- or postnatally [3, 4, 7, 16, 17]. In particular, there appears to be no restriction of function of helper or cytotoxic T-cells [3, 5, 7, 8, 11], and immune reconstitution of the host progresses up to a full degree [7, 18].

Tolerance toward both host and donor is achieved in these chimeric patients. The immune immaturity of the host explains the lack of donor cell rejection that of the donor explains the lack of graft-versus-host disease (GvHD) induced by transplanted cells.

**Figure 1:**
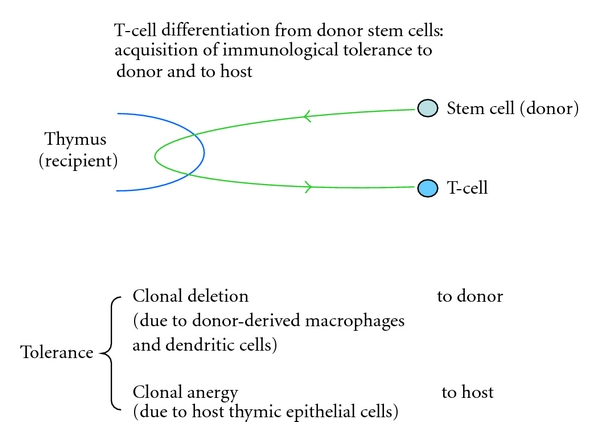
Differentiation of donor stem cells into mature T lymphocytes within the host thymus: acquisition of tolerance by thymocytes in contact with other donor cells and with host thymic epithelial cells.

Following SCT in our SCID patients, donor-reactive (but not host-reactive) cells have been shown to be deleted from the T-cell repertoire. Clonal deletion is therefore responsible for immunological tolerance to antigens of the donor and this process of negative selection is likely to occur in the host thymus, as a result of contact between thymocytes and dendritic cells or macrophages of donor origin (Figure 1).

In contrast, host-reactive T-cells (also designed as alloreactive T-cells since the stem cells that generated T-cells are allogeneic to other cells of the host body) remained present and relatively numerous in these chimeric patients. That no detrimental effect (GvHD or autoimmunity) occurs at any stage of T-cell development suggests that donor-derived T lymphocytes have been suppressed or anergized in the host. This hypothesis is supported by the specific absence of proliferative response to host stimulator cells in MLC. Further evidence of clonal anergy or suppression has been obtained in experiments involving transplantation of human fetal liver and thymus of similar or different origins in SCID mice [19]. Tolerance to host antigens appears to develop in human T-cells present in these experimental animals as the result of a clonal anergy that follows contact of human thymocytes with human epithelial cells of the host thymus fragment.

Transplantation tolerance is therefore induced by two different mechanisms: tolerance to donor by clonal deletion and tolerance to host by clonal anergy (Figure 1).

In nonimmunodeficient fetuses, tolerance was also apparently induced but, in contrast with SCID patients, the number of donor cells did not expand significantly over the years. Various hypotheses may account for this limited development of donor cells: selective advantage of host stem cells over donor stem cells, lack of “space” in the hematopoietic niches, allogeneic reactions from the progressively immunomature lymphocytes of the host, and allogeneic reactions from the maternal T-cells that have been shown to reduce engraftment after in utero SCT [20]. When allogeneic and autologous SCT in fetal sheep were compared, however, no significant difference was found between the two groups [21]. Low engraftment did not appear to result mostly from major histocompatibility complex-driven allogeneic reactions but rather from donor/host competition of another kind. Nevertheless, since donor-specific tolerance induction requires relatively minimal engraftment [18], clinical application may take advantage of such a tolerance produced at the fetal age to procure further treatment with allogeneic cells of similar origin, in larger numbers and with appropriate adjunctive treatment, later on in life.

## Figures and Tables

**Table 1:** HLA phenotypes of host cells and of cells of donor origin found in three chimeric patients.

Patient	Host/donor cells	HLA		
		A	B	DR
1	Host	3–33	14–47	4–11
	Donor	1-2	8–18	3–9
2	Host	2–31	37–62	4-5
	Donor	2–30	8–35	6-7
3	Host	2-3	27–44	4–14
	Donor	26–29	35–60	1–13

Reproduced from [7].

**Table 2:** Proliferative response of PBL from 2 patients and a normal donor to host, parental, and allogeneic cells.

Patient	Stimulator cells from					
	Medium	Host	Mother	Father	Allogeneic	Allogeneic
1	0.6 ± 0.0	1.4 ± 0.3	64.7 ± 3.5	40.0 ± 5.8	54.3 ± 1.2	75.6 ± 5.6
2	1.7 ± 0.3	4.1 ± 0.1	12.1 ± 0.4	ND	24.6 ± 2.0	22.4 ± 2.2
Normal donor (control)	2.1 ± 0.5	38.6 ± 1.1	22.7 ± 0.6	ND	47.2 ± 3.7	36.0 ± 2.9

The indicated data are cpm × 10^−3^ [^3^H] TdR incorporation expressed as the mean ± SD.

Reproduced from [12].
